# Supplementary material for: The impact of variance in carnitine palmitoyltransferase-1 expression on breast cancer prognosis is stratified by clinical and anthropometric factors
Source: PLoS One. 2023 Feb 3;18(2):e0281252. doi: 10.1371/journal.pone.0281252 (PMC9897541; doi:10.1371/journal.pone.0281252)
Supplement: Attachment — Submitted filename: Response to Reviewers PONE-D-22-26128.docx [file pone.0281252.s001.docx]

We thank the editors and reviewers for their time spent on this manuscript. Below please find our responses to all comments, queries, and concerns.

**Journal comments:**

**1. Please ensure that your manuscript meets PLOS ONE's style requirements, including those for file naming.**

Thank you for providing the links to the style templates. We have followed these requirements carefully.

**2. Thank you for stating the following in the Acknowledgments Section of your manuscript:**

**“This study was supported in part by a Pilot Grant from the Yale Cancer Center, and by a dkNET Summer of Data student fellowship. The funders had no role in study design, data collection and analysis, decision to publish, or preparation of the manuscript. The authors declare that no conflicts of interest exist.”**

**We note that you have provided additional information within the Acknowledgements Section that is not currently declared in your Funding Statement. Please note that funding information should not appear in the Acknowledgments section or other areas of your manuscript. We will only publish funding information present in the Funding Statement section of the online submission form.**

**Please remove any funding-related text from the manuscript and let us know how you would like to update your Funding Statement. Currently, your Funding Statement reads as follows:**

**“This study was supported in part by a Pilot Grant from the Yale Cancer Center, and by a dkNET Summer of Data student fellowship. The funders had no role in study design, data collection and analysis, decision to publish, or preparation of the manuscript.”**

**Please include your amended statements within your cover letter; we will change the online submission form on your behalf.**

The funding statement is correct as written. We have now removed any funding-related text from the manuscript.

**3. In your Data Availability statement, you have not specified where the minimal data set underlying the results described in your manuscript can be found. PLOS defines a study's minimal data set as the underlying data used to reach the conclusions drawn in the manuscript and any additional data required to replicate the reported study findings in their entirety. All PLOS journals require that the minimal data set be made fully available. For more information about our data policy, please see** [**http://journals.plos.org/plosone/s/data-availability**](http://journals.plos.org/plosone/s/data-availability)**.**

**Upon re-submitting your revised manuscript, please upload your study’s minimal underlying data set as either Supporting Information files or to a stable, public repository and include the relevant URLs, DOIs, or accession numbers within your revised cover letter. For a list of acceptable repositories, please see http://journals.plos.org/plosone/s/data-availability#loc-recommended-repositories. Any potentially identifying patient information must be fully anonymized.**

**Important: If there are ethical or legal restrictions to sharing your data publicly, please explain these restrictions in detail. Please see our guidelines for more information on what we consider unacceptable restrictions to publicly sharing data: http://journals.plos.org/plosone/s/data-availability#loc-unacceptable-data-access-restrictions. Note that it is not acceptable for the authors to be the sole named individuals responsible for ensuring data access.**

**We will update your Data Availability statement to reflect the information you provide in your cover letter.**

We have now provided links to the original data sets in the Materials and Methods section as well as in the Data Availability statement.

**4. Please ensure that you refer to Figure 3 in your text as, if accepted, production will need this reference to link the reader to the figure.**

In the revised version, we have ensured that all figures are referenced in the Results section.

**5. Please upload a new copy of Figure 6 as the detail is not clear. Please follow the link for more information: https://blogs.plos.org/plos/2019/06/looking-good-tips-for-creating-your-plos-figures-graphics/"** [**https://blogs.plos.org/plos/2019/06/looking-good-tips-for-creating-your-plos-figures-graphics/**](https://blogs.plos.org/plos/2019/06/looking-good-tips-for-creating-your-plos-figures-graphics/)

A new copy of Figure 6 has now been uploaded. We hope that it will now meet the technical requirements of the journal.

**6. Please review your reference list to ensure that it is complete and correct. If you have cited papers that have been retracted, please include the rationale for doing so in the manuscript text, or remove these references and replace them with relevant current references. Any changes to the reference list should be mentioned in the rebuttal letter that accompanies your revised manuscript. If you need to cite a retracted article, indicate the article’s retracted status in the References list and also include a citation and full reference for the retraction notice.**

The reference list has been reviewed to ensure that it is complete and correct.

**Reviewer comments:**

**Reviewer 1: Ryan Liu and authors in the manuscript “The Impact of Variance in Carnitine Palmitoyltransferase-1 Expression on Breast Cancer Prognosis is Stratified by Clinical and Anthropometric Factors” addressed the connection between breast cancer prognosis and fatty acid metabolism in cancer patients. CPT1A transports long-chain fatty acid to mitochondria for beta-oxidation and gene expression of this enzyme in the breast cancer tissue was used as a measurement of fatty acid metabolism rate within the cancer. To study this relationship, authors worked with available public databases and archive such as RNA-seq data and 18F-fluorothymidine PET-CT images and analyzed the data in relation to obesity and menopausal status using appropriate statistical methods. It is very interesting study, and their findings have significant impacts in the cancer metabolism field. In addition, the novelty of work is that authors skillfully combined research tools such as Fiji ImageJ, Xena Functional Genomics Explorer, MARRVEL, and GTEx Portal.**

We thank the reviewer for considering this a “very interesting study,” and asserting that “the novelty of [this] work is that [we] skillfully combined research tools such as Fiji ImageJ, Xena Functional Genomics Explorer, MARRVEL, and GTEx Portal.” We are delighted that this reviewer considers this a “very interesting study, and [our] findings have significant impacts in the cancer metabolism field.”

**Specific Points**

**1. 10.83 FPKM is used as cut off level for CPT1A gene expression. How did authors determine this cut off level? It was not explained in the Method section.**

The reviewer makes a great point, and the manuscript added the explanation: “10.83 FPKM was the cutoff level for CPT1A gene expression, as the samples were divided at the median.” The protocol in UCSC Xena determined this.

**2. Figures need to be referred in the Results, not in the Discussion.**

This helpfully suggested change has been made. We have removed all figure descriptions from the Discussion and only reference figures when necessary to convey the point expanded upon in the Discussion.

**3. In the sentence, the results on Figure 1A seems to be described in opposite way than the actual data.**

We appreciate the reviewer for pointing out this mistake. It has been corrected: “Patients with a high expression level of CPT1A had a lower survival rate (medial survival time of 3,500 days) than those with low expression levels of CPT1A (medial survival time of 4,200 days).”

**4. Figure 4 on Page 6 and Figure 5 on Page 7 need to be described better and correct way. For these data set, could age rather than menopausal status play a role?**

We thank the reviewer for pointing out these errors. They have been corrected: “Pre-menopausal breast cancer patients with a high expression level of CPT1A had a higher survival rate than their post-menopausal counterparts” and “Pre-menopausal breast cancer patients with a high expression level of CPT1A observed a higher survival rate than their post-menopausal counterparts until the two group’s intersection at 3663 days.”

We agree with the reviewer that age could play a role in the outcomes in this data set. We are not able to specifically test this question because of limited available data, but have added a comment on this important point in the Discussion:

“It should also be noted that our data on the interaction of menopausal status and BMI on breast cancer prognosis cannot distinguish between effects of age and of hormones *per se*. As aging is associated with increased ectopic lipid accumulation and increased *de novo* lipogenesis even independent of body weight, the effect of age could certainly confound the findings of the current study. However, due to the limited data available, we were not able to assess the impact of age as a continuous variable on the interaction between BMI, *CPT1*, and breast cancer outcomes.”

**5. In Figure 6, lower CPT1A expression in brain parts is not surprising, because CPT1C is the major isoform in brain. However, the highest expression of CPT1A is observed in GI-tract (colon, duodenum, and small intestine). Can authors add these tissue in the Figure 6 in other to compare with breast tissue?**

This is an important point that has been added in Figure 6. Breast tissue expression of CPT1A is now compared with blood, colon, kidney, liver, pancreas, small intestine, and stomach tissue.

**6. On page 9, the statement “these results are consistent with the fact that CPT1A is a major regulator of breast cancer” needs to be re-written. To make this statement, CPT1A activity assay or at least protein expression need to be determined. Authors for their interpretation, used only RNA-seq data and these data or actual gene expression of CPT1A is not confirmed by quantitative PCR in the tissue. Since metabolism in breast cancer cells not fully understood yet, a statement such as “a major regulator” should be avoided.**

The reviewer brings up a valid point. We have replaced the claim with a less definitive statement: “Figure 1b is consistent with the idea that CPT1A drives breast cancer metabolism.”

**7. On page 2, reference 3 was cited as “we demonstrated…” However, these authors were not listed as authors in the #3 manuscript. Please clarify this reference.**

This helpful suggestion has been incorporated, by removing “we demonstrated” and replacing it with “as demonstrated in the literature.”

**8. On page 2, “understanding” should be replaced by “understand”.**

This helpful correction has been made.

**9. In the Figure 2, should it be simpler and easier to understand if words “normal healthy, overweight and obese” included instead of 2,3,4 numbers?**

The reviewer makes a great point. Figure 2 now has qualitative x-axis labels.

**10. Number of participants (n) should be added in the legends for Figure 2 and 3.**

This helpful suggestion has been incorporated.

**11. Tissue names in Figure 6 on Page 8 are too small and not readable. Can authors replace this with higher resolution figure?**

The figures have been replaced with their high-resolution counterparts.

**Reviewer 2: The authors analyzed the role of CPT1A differentially expression on Breast Cancer Prognosis concrning Clinical and Anthropometric Factors. The method is approperiatly designed and the manuscript is well presented. The results were validated using different measurement including CT scan and RNA-seq analysis.**

We thank Reviewer 2 for stating that “the method is appropriately designed and the manuscript is well presented.” We are pleased that the reviewer values the validation of the conclusions.

**Specific Points**

**1. The figures are fuzzy. vector-based or high quality figures are required for the final version.**

The figures have been replaced with their high-resolution counterparts.

**2. The authors may refer to recent breast cancer progression works in the literature such as PMID: 35205681 and PMID: 34997055.**

We have reviewed the useful literature mentioned, and thank the reviewer for the suggestion. These papers have now been cited in the Introduction.
